# Transcriptomic Analysis of *Toxoplasma* Development Reveals Many Novel Functions and Structures Specific to Sporozoites and Oocysts

**DOI:** 10.1371/journal.pone.0029998

**Published:** 2012-02-13

**Authors:** Heather M. Fritz, Kerry R. Buchholz, Xiucui Chen, Blythe Durbin-Johnson, David M. Rocke, Patricia A. Conrad, John C. Boothroyd

**Affiliations:** 1 Department of Pathology, Microbiology and Immunology, University of California Davis, School of Veterinary Medicine, Davis, California, United States of America; 2 Department of Microbiology and Immunology, Stanford University School of Medicine, Stanford, California, United States of America; 3 Division of Biostatistics, University of California Davis, School of Medicine, Davis, California, United States of America; University of Georgia, United States of America

## Abstract

Sexual reproduction of *Toxoplasma gondii* occurs exclusively within enterocytes of the definitive felid host. The resulting immature oocysts are excreted into the environment during defecation, where in the days following, they undergo a complex developmental process. Within each oocyst, this culminates in the generation of two sporocysts, each containing 4 sporozoites. A single felid host is capable of shedding millions of oocysts, which can survive for years in the environment, are resistant to most methods of microbial inactivation during water-treatment and are capable of producing infection in warm-blooded hosts at doses as low as 1–10 ingested oocysts. Despite its extremely interesting developmental biology and crucial role in initiating an infection, almost nothing is known about the oocyst stage beyond morphological descriptions. Here, we present a complete transcriptomic analysis of the oocyst from beginning to end of its development. In addition, and to identify genes whose expression is unique to this developmental form, we compared the transcriptomes of developing oocysts with those of *in vitro*-derived tachyzoites and *in vivo*-derived bradyzoites. Our results reveal many genes whose expression is specifically up- or down-regulated in different developmental stages, including many genes that are likely critical to oocyst development, wall formation, resistance to environmental destruction and sporozoite infectivity. Of special note is the up-regulation of genes that appear “off” in tachyzoites and bradyzoites but that encode homologues of proteins known to serve key functions in those asexual stages, including a novel pairing of sporozoite-specific paralogues of AMA1 and RON2, two proteins that have recently been shown to form a crucial bridge during tachyzoite invasion of host cells. This work provides the first in-depth insight into the development and functioning of one of the most important but least studied stages in the *Toxoplasma* life cycle.

## Introduction

*Toxoplasma gondii* is an important zoonotic parasite that can infect a wide range of warm-blooded animals, including humans, with sometimes serious sequelae [1]–[3]. Like other Apicomplexa, *Toxoplasma* has a complex life cycle, in this case involving asexual replication in almost any warm-blooded animal and sexual reproduction only in felines. The latter culminates in the shedding of oocysts into the environment where they mature and persist as highly infectious forms. Infection of humans can result either from the eating of undercooked meat containing the asexual bradyzoite cyst stage or ingestion of mature oocysts as environmental contaminants of water or vegetables [4]. The relative importance of each route of exposure is not known as the methods to distinguish between tissue cyst and oocyst infection are still being developed [5], [6]. Nevertheless, epidemiologic studies support an important role for oocysts in transmission: the prevalence of toxoplasmosis is not reduced in vegetarians [7] and outbreaks tied to the ingestion of contaminated water have been reported globally, [8]–[12].

Oocysts are the product of a complex sexual reproduction that begins with ingestion by a feline of an infected prey [13], [14]. The encysted bradyzoites are released during digestion and these initiate a complex sexual development within the enterocytes of the cat's small intestine. After fertilization of a macrogamete by a microgamete, a zygote is formed and this is shed into the intestinal lumen as an immature oocyst about 3–7 days after ingestion of the infected prey. This is a highly efficient process with a single cat able to shed as many as one billion oocysts during a primary infection [13], [15], [16]. Upon defecation, the immature oocysts are released into the environment where they undergo a complex developmental process that starts with a single, relatively amorphous zygote and ends, after exposure to appropriate environmental conditions, with 8 discrete sporozoites subcompartmentalized within two sporocysts. Mature oocysts have been reported to survive and remain infective for years in fresh water [17] and for at least twenty-four months in salt water [18]. Their extreme resistance to treatments such as bleach, acid and ultraviolet makes them an important public health challenge. This also poses an interesting biological question: how can such a complex developmental process as sporulation occur within such an environmentally self-contained cyst?

Once ingested into an intermediate host, the wall of the mature oocyst must be ruptured and the sporozoites within must initiate a new infection by invading into intestinal epithelial tissue. Relatively little is known about both these processes although the sporozoite appears to have all of the organelles that recent work has shown are key to invasion by the asexual tachyzoites, i.e., micronemes, dense granules and rhoptries [19], [20]. Interestingly, invasion by sporozoites appears to involve a two-step process that includes formation of a spacious primary vacuole from which the sporozoite then escapes by formation of a tighter vacuole that superficially appears more similar to the one formed by an invading tachyzoite [21]. The molecular details of this complex invasion process are largely unknown. Similarly, almost nothing is known about how the sporozoite interacts with the infected host cell, especially compared to the recent detail that has emerged about the many virulence factors that tachyzoites introduce into the host cell during invasion [22]. Several dense granule proteins have been shown to be common to tachyzoites and sporozoites [21] but all such work has been dependent on having antibody reagents for the tachyzoite proteins as a starting point.

With recent advances in genome sequencing, a whole genome approach has provided a new way to discover and interrogate genes/proteins/pathways involved in a variety of processes, such as pathogenesis of disease, growth, adaptation, stress, and host-pathogen interactions. Among the most powerful methods that have been developed are microarrays to examine expression profiles for the entire transcriptome of a given organism. Such studies have markedly advanced our understanding of the biology of multiple organisms and disease states by providing information on gene regulation across different conditions and life stages. The *Toxoplasma gondii* genome sequence predicts ∼8,000 genes and an Affymetrix microarray with probesets representing all predicted genes (based on ToxoDB release 4) was recently developed and made commercially available [23]. Prior to the availability of this gene chip, most studies looking at gene expression in *T.gondii,* including developmental regulation during asexual development, utilized smaller scale custom microarrays [24]–[27], reverse transcription polymerase chain reaction (RT-PCR) [28], [29], expressed sequence tag (EST) analysis [30] or serial analysis of gene expression (SAGE) [31].

The goal of the current study was to understand the complex development of oocysts and gain insights into the functioning of the sporozoites within. The approach we chose was to use the *Toxoplasma* gene chip to characterize the transcriptome of oocysts throughout development and compare this to the expression profiles of the better-characterized tachyzoite and bradyzoite stages. Several groups of abundantly expressed genes were identified as up-regulated during oocyst development, including many that provide clues to their environmental resistance and to other functions, such as sporozoite invasion, that are specific to this key developmental stage.

## Results

**Figure 1:**
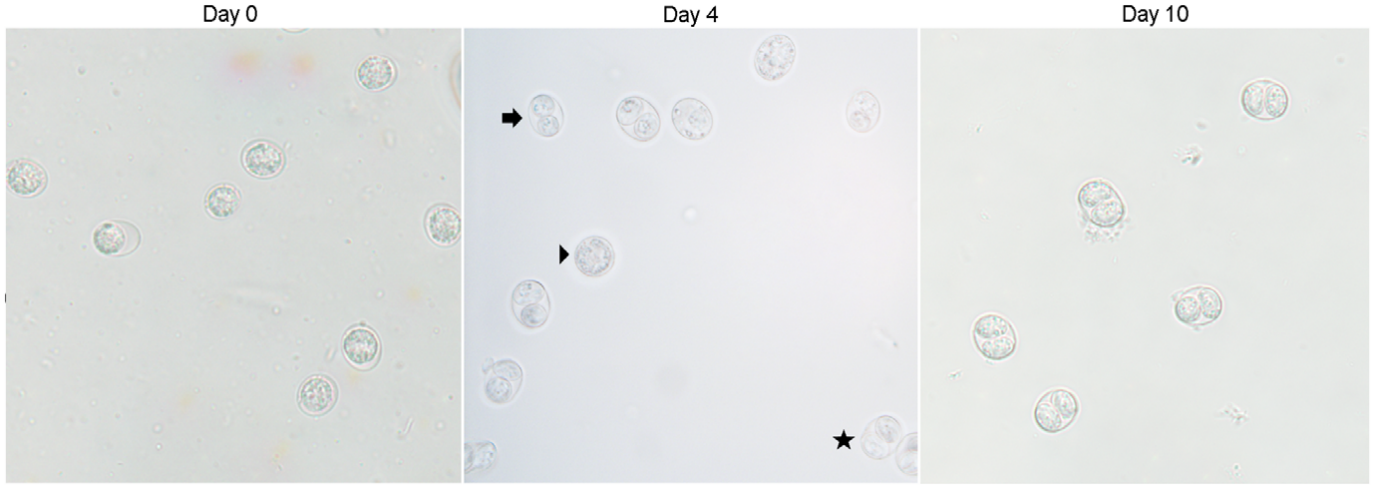
*Toxoplasma gondii* oocyst preparations used for transcriptomic analysis. Oocysts were harvested from cat feces as described in the materials and methods and isolated directly (d0; unsporulated) or after 4 (d4; mid-sporulation) or 10 (d10; sporulated) days of exposure to sporulation conditions. Day 0 oocysts are unsporulated with a primary sporoblast. Day 4 samples include unsporulated (arrowhead), partially-sporulated (star) and fully mature oocysts (arrow). Day 10 samples contain mature, sporulated oocysts.

Given our goal of interrogating gene expression across both the sexual and asexual stages of *Toxoplasma* development, it was important to have a strain capable of efficiently completing the entire life cycle. For all this work, we used the M4 strain isolated from an infected sheep in Scotland and kindly provided by Lee Innes of the Moredun Institute. Based on its European origin and the fact that, at each of 4 polymorphic loci, it was found to have a DNA sequence identical to that of the canonical Type II ME49 strain (see materials and methods), it is assumed that M4 is a type II strain. The oocysts used in this study were isolated from the feces of experimentally infected kittens within the first 24 hours of being shed. This material was also incubated for 4 and 10 days after shedding in conditions that allow oocyst maturation; these three time points represent immature (day 0), maturing (day 4) and mature (day 10) stages of oocyst development, respectively. Immature oocysts have yet to develop individual sporocysts or sporozoites; maturing organisms generally have recognizable sporocysts but few if any discernable sporozoites within those sporocysts; mature stages mostly have the full complement of 8 sporozoites subcompartmentalized as 4 organisms in each of two fully developed sporocysts (Figure 1). Using light microscopy to visualize oocysts on a hemocytometer, approximate percent sporulations were obtained. Day 0 oocysts were 100% unsporulated with only a primary sporoblast visually detected (d0, Figure 1). In the duplicate d4 samples, 52% and 78% of the counted oocysts had two distinct secondary sporoblasts/sporocysts, and of these, less than half had discernable sporozoites within (d4, Figure 1). In the duplicate day 10 oocyst samples, 95% and 99% of the oocysts had two sporocysts with a vast majority containing discernable sporozoites (day 10, Figure 1).

For comparison purposes, we used tachyzoites of the same M4 strain cultured *in vitro* by growth on human foreskin fibroblasts (HFFs) and bradyzoite-containing tissue cysts harvested from the brains of mice 3 weeks after oral infection with oocysts.

Preparation of RNA from oocysts requires first rupturing the oocyst wall which is, probably as a result of evolutionary selection, very resistant to most treatments. For this, we used a French press, similar to a previously described method [32]. The result was a relatively low yield, (about 1.4 µg in the d4 and d10 preparations and ∼10 µg in the d0 preparations, each from ∼5×10^6^ oocysts), but this was more than sufficient for the microarray analysis. For the bradyzoites, tissue cysts were prepared from infected mouse brain 21 days after infection. This time point was chosen because the numbers of cysts in the brain are near their peak and animals do not survive much beyond this time using the combination of infectious load, parasite strain and mouse strain employed here. In fact, to enable adequate tissue cyst numbers to be obtained and to prevent premature death of the animals, it was necessary to provide a low dose of sulfadiazine in the drinking water. Tachyzoites presented their own challenge with this strain as it grows considerably slower in culture than the usual laboratory strains, even other type II strains (data not shown). Nevertheless, sufficient material was easily generated for ample RNA preparation by growth of tachyzoites in human foreskin fibroblasts.

Duplicate RNA samples from all five stages, tachyzoites, bradyzoites and the three developmental stages of oocysts, were prepared and used as the starting material for transcriptomic analysis using the Affymetrix *Toxoplasma* gene chip [23]. To evaluate differences in transcript abundance across the sample groups, the probesets with corresponding version 5 gene IDs (ToxoDB.org v6.4) that were found to meet statistical criteria for differing levels of expression for each comparison group (p<0.05) were assembled. The tables report the mean of the generalized logarithm (glog) expression values for each duplicate sample and fold-changes in expression levels between different pairs of developmental stages. To estimate the background levels of expression, the median glog value was calculated for each sample group for 14 control probesets, encoding the luciferase, DsRed, Green Fluorescent Protein (GFP), ZsGreen, ZsYellow and chloramphenicol acetyl transferase (CAT) genes, for which no transcript should exist in our samples. The median range of expression for this control set was 3.1–3.2 across sample types and this, therefore, represents the approximate background value. A table showing the corresponding percentiles of expression for glog values is provided as a supplemental table (Table S1). Details of data normalization, glog transformation and the method used to calculate fold-changes are provided in the materials and methods and prior publications [33]–[35]. The complete list of all probe-sets with significant comparisons is provided as a supplemental table (Table S2). An in depth analysis and validation of the bradyzoite dataset as compared to the tachyzoites (only) is the subject of a separate report [36].

### Validation of stage-specific gene expression

A limited set of developmentally regulated genes has previously been identified for tachyzoites and bradyzoites and a more limited number in mature oocysts. To determine if these sporozoite-, tachyzoite- and bradyzoite-specific genes are regulated in these datasets in the way expected, a sample of the best-studied such genes was analyzed (Table 1). These include the canonical bradyzoite genes enolase 1 (*ENO1*), lactate dehydrogenase 2 (*LDH2*), bradyzoite antigen 1 (*BAG1*) and two surface antigen-1-related sequences (SRSs), *SRS9* and *SAG2X* [35], [37]–[39]. Canonical tachyzoite genes include *ENO2*, *LDH1* and the surface antigen genes *SAG1* and *SAG2A* [40]–[44]. RT-PCR and SAGE have identified differentially expressed genes in oocysts as compared to tachyzoites and bradyzoites, including those encoding the sporozoite-specific surface antigen, SporoSAG, and two putative oocyst wall proteins (TgOWPs), which are homologues to known *Cryptosporidium* OWPs (COWPs) [31], [45], [46]. Table 1 presents the mean normalized (glog) expression values across the samples and the fold-changes in the comparisons. Expression profiles in this dataset were consistent with previous descriptions of stage-specific transcripts confirming that the biological material, RNA preparation methods and microarray analyses used here are providing a faithful representation of the developmental forms under study, albeit with certain caveats due to technical limitations discussed further, below.

**Table 1:** Validation of gene expression comparing known developmentally-regulated *Toxoplasma gondii* genes.

			Mean glog expression (SEM)				
Regulation	Gene ID^1^	Product^2^	d10^3^	Tz^4^	Bz^5^	Fold-change * [fold-change lower]	
**Higher in Tachyzoites**						**Tz vs. d10**	**Tz vs. Bz**
	33460	SRS29B (SAG1)	8.4 (0.02)	9.3 (0.07)	4.6 (0.02)	-	46.0
	68850	ENO2	5.3 (0.08)	7.4 (0.09)	4.2 (0.10)	4.9	8.0
	32350	LDH1	6.9 (0.02)	6.0 (0.08)	5.1 (0.28)	[2.0]	1.8
	71050	SAG2A (SRS34A)	6.4 (0.01)	8.4 (0.09)	6.0 (0.06)	6.0	8.4
**Higher in Bradyzoites**						**Bz vs. d10**	**Bz vs. Tz**
	59020	BAG1	3.6 (0.54)	6.2 (0.18)	10.0 (0.01)	125.0	34.5
	68860	ENO1	2.7 (0.06)	5.2 (0.16)	9.4 (0.03)	76.9	35.7
	91040	LDH2	3.1 (0.14)	4.9 (0.25)	9.2 (0.03)	58.8	34.5
	120190	SRS9 (SRS16B)	3.3 (0.06)	4.1 (0.15)	5.6 (0.10)	2.6	2.1
	7140	SAG2X (SRS49B)	2.4 (0.20)	4.7 (0.03)	6.6 (0.09)	6.3	3.4
**Higher in Day 10 Oocysts**						**d10 vs. Tz**	**d10 vs. Bz**
	58550	SRS28 (SporoSAG)	9.5 (0.02)	4.5 (0.17)	4.1 (0.05)	55.6	63.2
	9610	oocyst wall protein COWP, putative (TgOWP2)	8.8 (0.02)	3.3 (0.19)	3.9 (0.13)	38.5	34.0
	48730	oocyst wall protein, putative (TgOWP5)	7.5 (0.04)	3.9 (0.12)	4.6 (0.07)	10.0	7.8

Mean normalized, glog-transformed expression values and calculated fold-change in expression levels for each pair-wise comparison. The standard error of the mean (SEM) is given in parentheses.

1 TGME49_Gene identifier according to ToxoDB.org (v6.4).

2 Identity of protein assigned by ToxoDB.org (v6.4).

3 Day 10 sporulated oocysts.

4 *in vitro*-derived tachyzoites (2 dpi).

5 *in vivo*-derived bradyzoites (21 dpi).

*Fold change calculated from glog mean expression values back transformed to the original scale (see materials and methods) and shown only where values are significantly different, p<0.05.

### Patterns of gene expression across oocyst maturation time points

As the oocyst develops from the unsporulated, freshly excreted form to a fully sporulated and infectious stage, significant changes are taking place. The sporocyst walls are formed and the oocyst accrues its full infectious potential through sporozoite formation. It was therefore expected that the developing oocyst would be transcriptionally active and that by looking at the gene-expression patterns at three stages in its development, unique patterns would be revealed and that these would provide clues to developmentally-regulated genes in the oocyst that are relevant to its maturation and environmental stability. For this analysis, comparisons were made between the transcriptomes of d4 versus d0 oocysts, d10 versus d0 oocysts and d10 versus d4 oocysts. In the three comparisons made, the greatest number of genes with significantly differing transcript levels occurred between d4 and d0 (2,362 significant genes) followed by d10 versus d0 (2,233 significant genes), with comparatively fewer significant genes found to differ in the d10 versus d4 comparison group (830 genes). Comparisons between d10 oocysts and the tachyzoite and bradyzoite stages identified 1,850 and 1,771 genes as significantly different in their expression, respectively (Table 2).

**Table 2:** Summary of numbers of genes with significantly differing levels of gene expression in pair-wise comparisons of oocyst maturation time points and between mature day 10 oocysts and tachyzoites and bradyzoites.

Comparison	Total number of significant genes	Up regulated in comparison	Down regulated in comparison
day 4 vs. day 0 oocysts	2362	938	1424
day 10 vs. day 0 oocysts	2233	889	1314
day 10 vs. day 4 oocysts	830	431	399
day 10 oocysts vs. tachyzoites	1850	1022	828
day 10 oocysts vs. bradyzoites	1771	929	842

Lists of the top 30 genes that were found to be significantly changed in their expression levels (either higher or lower) in each of the oocyst time-point comparisons were generated and are provided as a supplemental table (Table S3). As predicted, a number of genes related to sporozoite development were found to increase in transcript abundance in the d4 oocysts compared to d0. Within the top 30 list of significant changes showing the largest fold-increases in d4 compared to d0 oocysts were genes encoding: 13 hypothetical proteins, 3 of which were tyrosine-rich (defined as >5% tyrosine); 3 late embryogenesis abundant domain-containing proteins (LEAs); 3 micronemal proteins (MIC10, MIC 11 and MIC13); two SRS-family proteins (the previously described SporoSAG and SRS3); 2 dense granule proteins (GRA1 and GRA7); 2 PAN-domain-containing proteins; 2 antioxidant proteins (a putative glutathione/thioredoxin peroxidase and a superoxide dismutase, SOD3); a putative eukaryotic aspartyl protease; a putative serine protease inhibitor (TgPI-1); and a small heat shock protein 20 (Table S3). The timing of increased expression of secreted and surface-antigen-related proteins is coincident with the formation of sporozoites within the oocyst. Interestingly, an even greater number of genes had significantly lower transcript levels in d4 versus d0 oocysts, of which nearly half are hypothetical proteins. These may reflect genes involved in the later stages of macrogamete development and oocyst formation occurring in the feline intestine; i.e., genes that function in earlier stages of the parasite's sexual cycle (e.g., gamete formation and fertilization), stages that are difficult to obtain and that were not available for analysis in the current work.

**Figure 2:**
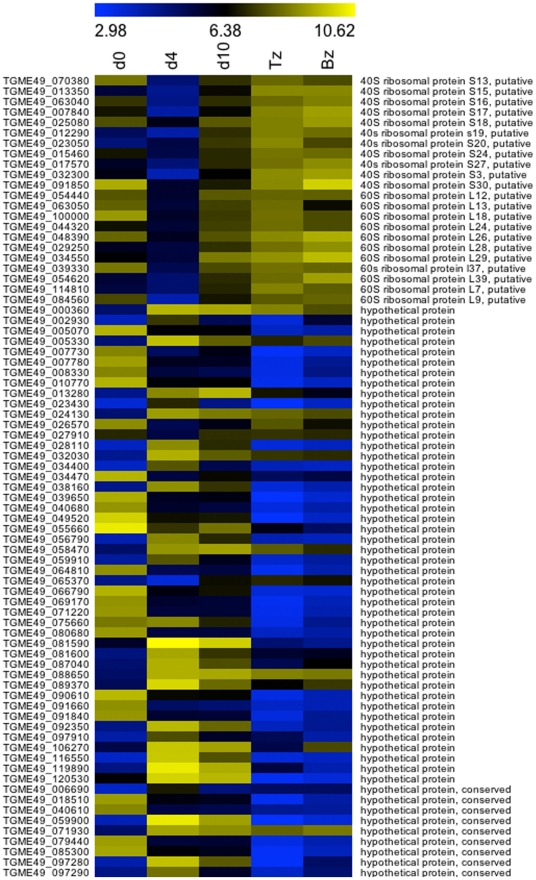
Heatmap of the average normalized expression values (glog) of ribosomal and hypothetical genes in the top 30 genes with significantly changing expression levels, higher and lower, across all pair-wise comparisons of oocyst time points. Expression values for tachyzoite and bradyzoite stages were included to highlight genes that appear to be oocyst-specific and those that resemble tachyzoites and/or bradyzoites in their transcript levels. The range of expression is represented by a color grade ranging from low (blue) to high (yellow).

**Figure 3:**
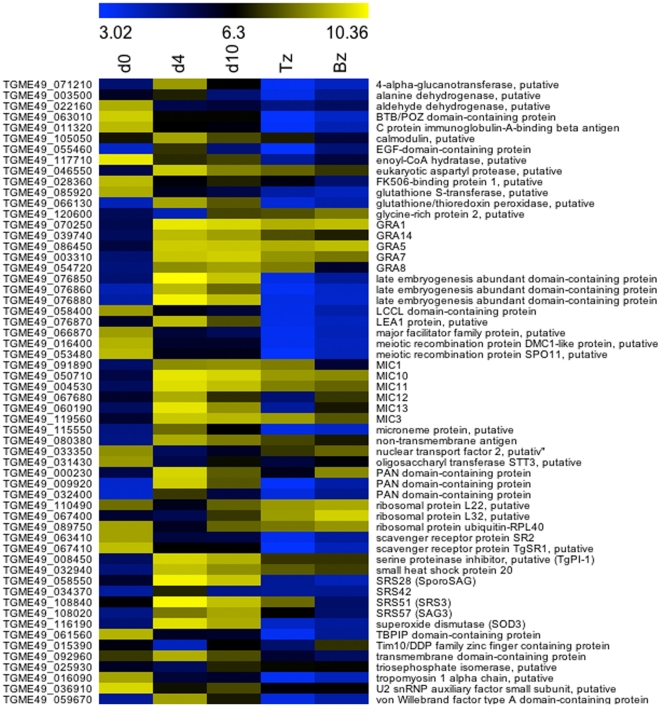
Heatmap of the average normalized expression values (glog) of the top 30 genes with significantly changing expression levels, higher and lower, across all pair-wise comparisons of oocyst time points (ribosomal and hypothetical genes not included, shown in **figure 2**). Expression values for tachyzoite and bradyzoite stages were included to highlight genes that appear to be oocyst-specific and those that resemble tachyzoites and/or bradyzoites in their transcript levels. The range of expression is represented by a color grade ranging from low (blue) to high (yellow).

A list that combines the top 30 changes from all of the pair-wise comparisons between the three oocyst time points, which includes 135 genes, is displayed as heat-maps (Figs. 2 and 3). Tachyzoite and bradyzoite expression levels are included for comparison. Within this list are a number of gene transcripts that were most abundant in the d0 oocyst compared to later oocyst, bradyzoite and tachyzoite stages. In addition to several hypothetical proteins, gene transcripts specifically elevated in d0 oocysts included two meiotic recombination proteins, (DMC1-like and SPO11), two scavenger receptor proteins (TgSR1 and SR2), a Tat-binding protein-1 interacting protein (TBPIP)-domain-containing protein, an aldehyde dehydrogenase, a glutathione S-transferase, an enoyl Co-A hydratase, a putative oligosaccharyl transferase STT3, a major facilitator family protein, an LCCL-domain-containing protein, a BTB/POZ-domain-containing protein (TGME49_063010), a U2 snRNP auxiliary factor small subunit, a C protein immunoglobulin-A-binding beta antigen, a putative tropomyosin 1 alpha chain, and a putative FK506-binding protein 1. In all pair-wise comparisons, a large number of the significantly changing genes were “hypothetical” proteins, which have not been characterized or been ascribed a putative function. These appear likely to have functions specific to one or other developmental form. The identification here of the stage in which they are most highly expressed will help provide clues to their function.

### Oocyst-specific transcripts

To identify genes specifically relevant to the mature oocyst, two comparisons were performed: d10 oocysts versus tachyzoites (tz) and d10 oocysts versus bradyzoites (bz). In addition to the transcriptomic analysis presented here, preparations of d10 oocysts were also processed for proteomic analysis, including separated wall and sporocyst fractions, as described in the accompanying manuscript [47]. While the focus of the proteomic work was specifically on the wall composition, and no proteomic data were generated for the earlier time points in development (i.e., d0 and d4 oocysts) or for tachyzoites or bradyzoites, the protein data do provide important validation for the expression of some of the novel, oocyst-specific genes discussed here. The gene lists to be discussed below, therefore, include an indication of whether there is proteomic evidence for each gene's expression in d10 oocysts and, using data present in ToxoDB (v6.4), in tachyzoites (several tachyzoite proteomic datasets can be found within ToxoDB and, for these purposes, we considered them in aggregate). Because the proteomic datasets that are being compared were generated in different labs using different methodologies, no attempt was made to draw quantitative conclusions about relative expression; hence, we simply scored a given protein as either detected or not. The criteria used to score a protein as detected in the oocyst proteome required a minimum identification of two unique peptides. No minimum criterion was set for tachyzoites, which were scored in the tables presented here as “detected” if any peptide identification for the given protein had been reported on ToxoDB. It is important to note that the absence of proteomic data for a given protein has only limited predictive value as there can be many technical reasons for failure to detect a protein. These data, then, are mostly useful for a positive result, i.e., to confirm a gene's expression. This is especially the case for the oocyst proteomic data where only one lab's efforts are available. For the tachyzoites, five independent datasets exist on ToxoDB and so a failure to detect a given protein in any of those five sets is a still imperfect but more likely indicator that there is little if any expression in that stage.

The comparisons of the transcriptomic data for d10 oocysts, tachyzoites and bradyzoites were assembled into a single list comprising the top 25 genes with the greatest fold-increase in d10 oocysts compared to both tachyzoites and bradyzoites (Table 3). Many of the genes in this comparison had even higher expression in d0 and/or d4 oocysts, but only the d10 transcriptome was compared to the transcriptomes of tachyzoites and bradyzoites. As a result, some of the genes listed here also appear on the list of genes with the highest expression in d0 oocysts. Included in the list here are genes encoding: 2 late-embryogenesis-domain-containing proteins (LEAs); 2 antioxidant proteins (glutaredoxin and superoxide dismutase - SOD3); one outer wall protein (TgOWP2); one haloacid dehalogenase-like protein; one oxidoreductase family protein (alanine dehydrogenase); one SRS-family protein (SporoSAG); and 16 hypothetical proteins, 5 of which are tyrosine-rich (>5% tyrosine). Several of these genes have been shown elsewhere to be specifically elevated in oocysts/sporozoites, including: *SRS28*/*SporoSAG* [45]; the putative *OWPs* [46]; *SOD3* and *glutaredoxin* (TGME49_027100) [48]; and one *LEA*, designated *TgERP* for embryogenesis-related protein and corresponding to TGME49_076850 [5]. Seven putative oocyst wall proteins have been identified in the ToxoDB, based on homology to *Cryptosporidium* OWPs (COWPs). Of these, one is in the top 25 list of significantly up-regulated genes in oocysts (TgOWP2; Table 3). Four LEAs are designated in the TGME49 genome, all of which were significantly up-regulated in oocysts compared to tachyzoites and bradyzoites and 2 of which were in the top 25 list. As can be seen in this Table, 18/25 (72%) of the genes that are strongly up-regulated in d10 oocysts compared to the two asexual stages also have proteomic data to indicate expression and only 12% have proteomic evidence for expression in tachyzoites. While these data are not quantitative, especially as regards the failure to detect a protein in the oocyst dataset, as discussed above they do provide strong corroborative support for the microarray data.

**Table 3:** Top 25 genes with significantly higher expression in day 10 oocysts compared to tachyzoites and bradyzoites, with day 0 and 4 oocysts included for comparison.

		Mean glog expression (SEM)					Fold-change *		Proteomic evidence	
Gene ID^1^	Product^2^	d0^3^	d4^3^	d10^3^	Tz^4^	Bz^5^	d10 vs. Tz	d10 vs. Bz	Oo^6^	Tz^7^
002100	hypothetical protein	11.4 (0.54)	10.1 (0.22)	9.9 (0.05)	3.5 (0.20)	3.7 (0.35)	111.1	103.3	Y	N
037080	hypothetical protein (6.2% Tyr)	11.2 (0.52)	9.6 (0.35)	9.6 (0.05)	3.6 (0.22)	3.7 (0.03)	90.9	84.6	Y	N
120530	hypothetical protein (5.6% Tyr)	6.3 (0.13)	9.9 (0.15)	9.4 (0.07)	3.3 (0.20)	3.6 (0.20)	76.9	69.7	Y	N
002110	hypothetical protein	11.0 (0.46)	9.0 (0.63)	9.4 (0.05)	3.1 (0.18)	3.8 (0.09)	76.9	63.8	N	N
076850	LEA (TgERP)	4.9 (0.07)	10.4 (0.13)	9.4 (0.02)	3.5 (0.31)	4.4 (0.05)	71.4	55.4	Y	N
076880	LEA	4.3 (0.13)	10.2 (0.14)	9.3 (0.02)	3.5 (0.03)	3.8 (0.04)	66.7	60.0	Y	N
081590	hypothetical protein (15.5% Tyr)	5.9 (0.01)	10.6 (0.07)	9.9 (0.08)	4.9 (0.04)	4.6 (0.28)	66.7	77.6	Y	N
120540	hypothetical protein	10.5 (0.65)	9.2 (0.28)	9.2 (0.04)	3.7 (0.01)	3.8 (0.11)	52.6	51.7	N	N
098560	hypothetical protein	6.3 (0.07)	9.5 (0.19)	9.1 (0.02)	3.09 (0.22)	3.9 (0.23)	58.8	48.0	N	N
059900	hypothetical protein, conserved	4.1 (0.02)	10.3 (0.08)	9.0 (0.11)	3.1 (0.11)	3.6 (0.02)	55.6	47.5	Y	Y
058550	SRS28 (SporoSAG)	5.7 (0.14)	10.2 (0.07)	9.5 (0.02)	4.5 (0.17)	4.1 (0.05)	55.6	63.2	Y	N
094600	hypothetical protein	6.7 (0.14)	9.5 (0.22)	9.2 (0.07)	3.7 (0.12)	4.3 (0.15)	52.6	43.9	Y	N
027100	glutaredoxin, putative	6.9 (0.67)	9.9 (0.15)	9.2 (0.05)	3.2 (0.02)	4.4 (0.17)	62.5	45.7	Y	N
119890	hypothetical protein (5.5% Tyr)	4.7 (0.29)	10.3 (0.04)	9.3 (0.04)	5.5 (0.13)	4.2 (0.04)	26.3	51.2	Y	N
087250	hypothetical protein (13.5% Tyr)	11.0 (0.44)	8.6 (0.73)	9.1 (0.02)	4.3 (0.01)	3.6 (0.09)	41.7	50.7	Y	N
066860	BTB/POZ domain-containing protein	10.9 (0.54)	8.3 (0.93)	9.1 (0.00)	5.6 (0.09)	3.8 (0.19)	21.7	48.2	N	N
004520	hypothetical protein	6.0 (0.02)	9.5 (0.31)	9.0 (0.17)	3.4 (0.10)	4.0 (0.14)	50.0	42.3	Y	Y
116190	superoxide dismutase, putative (SOD3)	4.9 (0.05)	9.9 (0.08)	9.0 (0.07)	3.8 (0.18)	4.4 (0.22)	45.5	37.8	Y	N
009610	oocyst wall protein COWP, putative (TgOWP2)	10.7 (0.44)	8.4 (0.74)	8.8 (0.02)	3.3 (0.19)	3.9 (0.13)	38.5	34.0	Y	N
070950	hypothetical protein	6.5 (0.03)	8.9 (0.45)	8.7 (0.04)	3.8 (0.28)	4.5 (0.18)	31.3	25.3	Y	N
029320	haloacid dehalogenase-like hydrolase domain-containing	10.2 (0.31)	7.9 (0.46)	8.5 (0.02)	3.4 (0.14)	3.8 (0.05)	29.4	26.2	N	N
072240	hypothetical protein	5.2 (0.03)	8.7 (0.34)	8.5 (0.01)	3.4 (0.15)	4.3 (0.06)	28.6	22.1	Y	N
002090	hypothetical protein	10.4 (0.20)	8.0 (0.440	8.5 (0.02)	3.4 (0.00)	3.7 (0.11)	28.6	26.3	N	N
115260	alanine dehydrogenase, putative	8.5 (0.02)	8.4 (0.17)	8.4 (0.03)	3.6 (0.22)	4.4 (0.39)	25.6	19.8	Y	N
005090	hypothetical protein	9.0 (0.34)	8.4 (0.59)	8.4 (0.02)	3.7 (0.14)	4.1 (0.39)	24.4	21.5	N	Y

Mean normalized, glog-transformed expression values and calculated fold-change in expression levels for each pair-wise comparison. The standard error of the mean (SEM) is given in parentheses.

1 TGME49_Gene identifier according to ToxoDB.org (v6.4).

2 Identity of protein assigned by ToxoDB.org (v6.4).

3 Oocysts sporulated for 0, 4 or 10 days.

4 *in vitro*-derived tachyzoites (2 dpi).

5 *in vivo*-derived bradyzoites (21 dpi).

6 Detection in day 10 (mature) oocyst proteome. Yes (Y) indicates that a minimum of 2 unique peptides mapping to given protein were identified by mass spectrometry.

7 Previous mass spectrometry evidence of expression in tachyzoites according to ToxoDB.org (v6.4).

*Fold-change calculated from glog mean expression values back transformed to the original scale (see materials and methods) and shown only where values are significantly different, p<0.05.

### Patterns of expression for genes encoding surface antigens and secreted proteins

*Toxoplasma* tachyzoites invade a host cell by attachment and active penetration followed by growth within a parasitophorous vacuole (PV). Many studies have shown the remarkable extent to which tachyzoites modify their intracellular environment, presumably to promote their own replication. Attachment, invasion and intracellular survival are apparently facilitated by the tachyzoite's surface antigens and the protein contents of their secretory organelles; micronemes (containing MICs), rhoptries (containing ROPs and RONs) and dense granules (containing GRAs) [49]. Much less is known about sporozoite invasion but *in vitro* studies have shown that the sporozoite temporarily occupies a primary PV before it moves into a second PV where the parasite replicates [21]. Virtually nothing is known about how the sporozoite interacts with the host cell at the molecular level. Given the possibility that the sporozoite utilizes a unique, two-step method to create the parasitophorous vacuole and the fact that there is essentially only one host cell type encountered by sporozoites (i.e., intestinal epithelial cells), it might be expected that the oocyst/sporozoite would have an unusual repertoire of surface antigens and secreted proteins. These then became the focus of our analysis.

#### Surface antigens

Developmental regulation of the SRS family of genes has been well described in tachyzoites and bradyzoites [24], [44]. The SRS proteins are designated as such based on their structural similarity to the major, immunodominant surface antigen SAG1 (also known as SRS29B or P30) which is abundantly found on the surface of tachyzoites and which was the first *Toxoplasma* surface antigen to have its cognate gene sequenced and complete protein structure determined [50], [51]. Although varied in their specific immunogenicity and function, SRS proteins are involved in host cell invasion at the level of attachment and recognition by the host cell (thereby influencing host immune response). The hallmark surface antigen in sporozoites is SRS28, or SporoSAG [45], but the surface antigen repertoire of the sporozoite is not limited to SporoSAG. Surface antigen genes previously shown to be expressed in both sporozoites and tachyzoites by SAGE and EST data include *SAG1*, *SAG3* and *SRS3* [31]. Those results were confirmed in the microarray data reported here; Table 4 shows a complete list of SRS genes with significant differences in expression levels between oocysts and one or other of the asexual developmental forms. Interestingly, the repertoire of *SRS* gene expression in oocysts more closely paralleled tachyzoites than bradyzoites; that is, many *SRS* genes that were up-regulated in bradyzoites relative to tachyzoites were generally “off” in oocysts (e.g., *SAG2C/D/X/Y, SAG4, SAG4.2 and SRS22A*), whereas several SRS genes that are up in tachyzoites relative to bradyzoites were also expressed at substantial levels in oocysts (e.g., *SAG1 and SRS3*). Surprisingly, *SAG3*, a SRS protein that is common to tachyzoites and bradyzoites [52] showed transcript levels that were ∼11- and 27-fold higher in d10 oocysts compared to tachyzoites and bradyzoites, respectively. This level of up-regulation is approaching that of the better-known *SporoSAG,* which showed 56- and 63-fold higher transcript levels in d10 oocysts relative to tachyzoites and bradyzoites, respectively. Both these genes showed much higher levels of expression in the d4 and d10 oocysts compared to d0, presumably reflecting induction as sporozoites begin to form. The proteomic data strongly corroborated the d10 oocyst microarray results: i.e., with one exception, there was a perfect correlation between having a glog expression value above 6 and being expressed (and there was no protein detected for genes with glog values below 6). The one exception was *SRS42*, which had a glog expression value of 4.6 but a positive detection in the d10 oocyst proteome. The correlation was less strong for the tachyzoite data but, as predicted, SAG3 but not SporoSAG, has been detected in tachyzoite proteomes.

**Table 4:** Expression of functionally interesting genes of *Toxoplasma gondii* oocysts at different stages of maturation with a comparison to bradyzoite and tachyzoite expression levels - SRS family proteins.

		Mean glog expression (SEM)					Fold-change * [fold-change lower]					Proteomic evidence	
Gene ID^1^	Product^2^	d0^3^	d4^3^	d10^3^	Tz^4^	Bz^5^	d4 vs. d0	d10 vs. d4	d10 vs. d0	d10 vs. Tz	d10 vs. Bz	Oo^6^	Tz^7^
058550	SRS28 (SporoSAG)	5.7 (0.14)	10.2 (0.07)	9.5 (0.02)	4.5 (0.17)	4.1 (0.05)	59.3	-	28.3	55.6	63.2	Y	N
108020	SAG3 (SRS57)	4.7 (0.21)	8.2 (0.28)	9.0 (0.02)	6.3 (0.03)	5.0 (0.06)	15.3	-	31.2	11.1	26.8	Y	Y
101150	SRS8 (SRS19B)	3.5 (0.20)	3.5 (0.25)	3.1 (0.07)	4.3 (0.13)	3.2 (0.09)	1.3	-	1.3	5.6	2.2	N	N
058810	SRS27B	4.3 (0.20)	7.9 (0.20)	6.4 (0.04)	3.5 (0.07)	4.5 (0.10)	13.0	[3.7]	3.6	4.4	3.2	Y	N
108840	SRS3 (SRS51)	6.2 (0.08)	10.3 (0.12)	9.4 (0.06)	8.0 (0.01)	5.0 (0.00)	43.7	[2.4]	17.9	3.8	38.9	Y	Y
119350	SRS domain containing protein	5.4 (0.11)	8.2 (0.31)	7.0 (0.04)	5.3 (0.14)	5.4 (0.11)	9.8	[3.0]	3.3	3.5	3.2	Y	Y
067130	SRS38A	4.0 (0.06)	7.0 (0.31)	5.5 (0.08)	4.4 (0.16)	6.2 (0.11)	6.5	[3.2]	2.1	1.8	-	N	Y
038440	SRS22A	4.5 (0.21)	4.5 (0.05)	4.7 (0.06)	3.9 (0.21)	9.3 (0.01)	-	-	-	1.3	[43.5]	N	N
033460	SAG1 (SRS29B)	5.4 (0.22)	8.0 (0.63)	8.4 (0.02)	9.3 (0.07)	4.6 (0.05)	8.3	-	13.1	-	19.0	Y	Y
115320	SRS52A	4.2 (0.08)	5.8 (0.50)	6.4 (0.04)	5.6 (0.14)	4.4 (0.16)	2.3	-	3.6	-	3.4	Y	Y
034370	SRS42	4.4 (0.17)	6.9 (0.04)	4.6 (0.07)	4.4 (0.12)	4.5 (0.02)	5.0	[4.9]	-	-	-	Y	N
024170	SRS domain-containing protein	3.9 (0.15)	3.5 (0.25)	3.0 (0.42)	4.0 (0.05)	7.0 (0.03)	-	-	-	-	[7.8]	N	N
007130	SAG2Y (SRS49A)	4.4 (0.13)	3.3 (0.00)	3.2 (0.10)	4.9 (0.15)	7.2 (0.11)	[1.4]	-	[1.4]	[1.7]	[9.1]	N	Y
007140	SAG2X (SRS49B)	3.7 (0.23)	3.0 (0.20)	2.5 (0.20)	4.7 (0.03)	6.6 (0.09)	-	-	[1.3]	[1.9]	[6.3]	N	Y
007160	SAG2C (SRS49D)	5.8 (0.36)	4.5 (0.36)	3.9 (0.35)	5.5 (0.15)	7.2 (0.01)	[2.2]	-	[2.5]	[2.0]	[7.9]	N	N
080580	SAG4.2	6.1 (0.15)	4.1 (0.25)	3.9 (0.20)	5.5 (0.08)	8.9 (0.00)	[2.8]	-	[3.0]	[2.0]	[38.5]	N	N
007150	SAG2D (SRS49C)	5.2 (0.37)	3.8 (0.03)	3.6 (0.19)	5.4 (0.32)	7.6 (0.02)	[1.8]	-	[1.9]	[2.1]	[12.1]	N	N
085870	SRS20A	4.1 (0.02)	4.8 (0.09)	4.8 (0.02)	7.2 (0.08)	6.1 (0.06)	1.3	-	1.3	[5.6]	[2.2]	N	Y
071050	SAG2A (SRS34A)	5.3 (0.40)	7.6 (0.38)	6.4 (0.01)	8.4 (0.09)	6.0 (0.06)	6.1	-	-	[6.0]	-	Y	Y
033480	SRS2 (SRS29C)	4.1 (0.04)	4.0 (0.31)	3.9 (0.11)	7.6 (0.24)	4.2 (0.12)	-	-	-	[11.1]	-	N	Y

Mean normalized, glog-transformed expression values and calculated fold-change in expression levels for each pair-wise comparison. The standard error of the mean (SEM) is given in parentheses.

1 TGME49_Gene identifier according to ToxoDB.org (v6.4).

2 Identity of protein assigned by ToxoDB.org (v6.4).

3 Oocysts sporulated for 0, 4 or 10 days.

4 *in vitro*-derived tachyzoites (2 dpi).

5 *in vivo*-derived bradyzoites (21 dpi).

6 Detection in day 10 (mature) oocyst proteome. Yes (Y) indicates that a minimum of 2 unique peptides mapping to given protein were identified by mass spectrometry.

7 Previous mass spectrometry evidence of expression in tachyzoites according to ToxoDB.org (v6.4).

*Fold change calculated from glog mean expression values back transformed to the original scale (see materials and methods) and shown only where values are significantly different, p<0.05.

#### Secreted proteins (MICs, ROPs, RONs and GRAs)

Microneme proteins (MICs) are associated with parasite motility and host cell invasion [53]. All but two microneme proteins that were generated in the list of significant comparisons between oocysts and the two asexual stages (>3-fold difference in at least one of the comparisons) had significantly increased expression in the d10 and d4 oocysts compared to d0, including *MIC1*, *MIC2*, *M2AP*, *MIC3*, *MIC4*, *MIC5*, *MIC10*, *MIC11*, *MIC12*, *MIC13*, *MIC16*, *AMA1*, an *AMA1* paralogue and a putative microneme protein (Table 5). Two genes encoding microneme proteins, *MIC13* and a putative microneme protein (TGME49_115550), had significantly higher transcript levels in day 10 oocysts compared to both bradyzoites and tachyzoites and may represent micronemal proteins most relevant to the oocyst/sporozoite stage. The proteomic data generally corroborated the microarray results with most MICs detected in both the oocysts and tachyzoites. The only micronemal proteins not detected in the tachyzoite proteomic data on ToxoDB were all in cases where the glog expression value was below 4 in tachyzoites.

**Table 5:** Functionally interesting genes of *Toxoplasma gondii* oocysts at different stages of maturation with a comparison to bradyzoite and tachyzoite expression levels – micronemes.

		Mean glog expression (SEM)					Fold-change * [fold-change lower]					Proteomic evidence	
Gene ID^1^	Product^2^	d0^3^	d4^3^	d10^3^	Tz^4^	Bz^5^	d4 vs. d0	d10 vs. d4	d10 vs. d0	d10 vs. Tz	d10 vs. Bz	Oo^6^	Tz^7^
060190	MIC13	5.1 (0.25)	10.0 (0.09)	8.6 (0.04)	4.4 (0.01)	6.7 (0.05)	67.2	[3.8]	17.7	24.4	6.1	Y	Y
067680	MIC12	5.7 (0.48)	9.0 (0.04)	7.0 (0.03)	4.9 (0.15)	7.2 (0.08)	18.7	[6.6]	2.8	4.5	-	Y	Y
115550	microneme protein, putative	4.7 (0.04)	8.0 (0.29)	6.3 (0.05)	3.5 (0.17)	3.8 (0.13)	12.1	[4.4]	2.7	4.0	3.7	Y	N
004530	MIC11	5.2 (0.10)	9.8 (0.06)	9.4 (0.04)	8.4 (0.06)	7.9 (0.07)	57.8	[1.6]	35.7	2.7	4.0	Y	Y
050710	MIC10	5.5 (0.13)	10.0 (0.08)	9.7 (0.13)	8.7 (0.02)	8.5 (0.05)	55.8	-	42.2	2.5	3.1	Y	Y
115540	microneme protein, putative	6.6 (0.44)	7.3 (0.33)	5.7 (0.08)	3.5 (0.12)	3.9 (0.05)	-	[3.7]	-	2.5	2.3	N	N
089630	MIC16	5.1 (0.24)	7.2 (0.07)	6.7 (0.05)	5.9 (0.18)	5.1 (0.12)	4.8	-	3.1	1.9	3.2	N	Y
115730	AMA1-paralogue [SporoAMA1]	3.8 (0.13)	6.1 (0.25)	5.1 (0.03)	3.6 (0.28)	3.6 (0.01)	3.2	[2.0]	1.6	1.7	1.7	Y	N
091890	MIC1	5.0 (0.26)	8.6 (0.05)	8.6 (0.11)	8.3 (0.06)	5.7 (0.11)	17.4	-	18.5	-	12.9	Y	Y
014940	M2AP	5.4 (0.51)	8.4 (0.12)	8.4 (0.09)	8.0 (0.18)	6.6 (0.05)	12.5	-	12.5	-	5.5	Y	Y
119560	MIC3	5.7 (0.20)	9.5 (0.10)	9.2 (0.07)	8.8 (0.08)	7.7 (0.10)	30.5	-	23.5	-	4.5	Y	Y
001780	MIC2	5.6 (0.31)	7.3 (0.31)	7.8 (0.13)	7.8 (0.05)	6.0 (0.02)	3.9	-	5.9	-	4.4	Y	Y
077080	MIC5	5.1 (0.31)	8.7 (0.31)	8.4 (0.03)	8.4 (0.05)	7.0 (0.03)	19.9	-	14.6	-	3.6	N	Y
055260	AMA1	4.8 (0.30)	7.5 (0.07)	7.3 (0.01)	7.1 (0.12)	6.1 (0.04)	7.4	-	6.2	-	2.7	Y	Y
008030	MIC4	4.1 (0.04)	6.4 (0.54)	6.9 (0.01)	7.3 (0.07)	6.5 (0.14)	3.7	-	5.5	-	-	Y	Y
008740	microneme protein, putative	4.3 (0.10)	4.3 (0.48)	4.2 (0.26)	6.6 (0.12)	7.5 (0.11)	-	-	-	[4.1]	[9.1]	N	Y

Mean normalized, glog-transformed expression values and calculated fold-change in expression levels for each pair-wise comparison. The standard error of the mean (SEM) is given in parentheses.

1 TGME49_Gene identifier according to ToxoDB.org (v6.4).

2 Identity of protein assigned by ToxoDB.org (v6.4).

3 Oocysts sporulated for 0, 4 or 10 days.

4 *in vitro*-derived tachyzoites (2 dpi).

5 *in vivo*-derived bradyzoites (21 dpi).

6 Detection in day 10 (mature) oocyst proteome. Yes (Y) indicates that a minimum of 2 unique peptides mapping to given protein were identified by mass spectrometry.

7 Previous mass spectrometry evidence of expression in tachyzoites according to ToxoDB.org (v6.4).

*Fold change calculated from glog mean expression values back transformed to the original scale (see materials and methods) and shown only where values are significantly different, p<0.05.

Rhoptries are secretory organelles that release their contents during host cell invasion. The rhoptry neck proteins (RONs) play a key role in host cell invasion. Several of the RONs collaborate with micronemal AMA1 to form the moving junction (MJ), a ring-like interface between the parasite and host plasma membranes that migrates down the length of the parasite during invasion [54]–[56]. The rhoptry bulb proteins (ROPs) appear to serve a downstream role, modifying the host-cell environment for the parasite's own purposes [57]–[59]. The microarray data yielded many significant changes in expression of rhoptry genes (Table 6). As seen with the SRS and micronemal genes, markedly increased transcript levels were observed for several ROP- and RON-encoding genes in d4 and d10 oocysts relative to d0.

**Table 6:** Functionally interesting genes of *Toxoplasma gondii* oocysts at different stages of maturation with a comparison to bradyzoite and tachyzoite expression levels - rhoptry proteins**.

		Mean glog expression (SEM)					Fold Change * [fold change lower]					Proteomic evidence	
Gene ID^1^	Product^2^	d0^3^	d4^3^	d10^3^	Tz^4^	Bz^5^	d4 vs. d0	d10 vs. d4	d10 vs. d0	d10 vs. Tz	d10 vs. Bz	Oo^6^	Tz^7^
009980	ROP42	4.6 (0.36)	7.9 (0.58)	7.2 (0.13)	3.8 (0.06)	7.6 (0.20)	11.8	-	6.1	7.9	-	Y	Y
114250	BRP1	5.1 (0.20)	8.4 (0.78)	7.7 (0.10)	5.0 (0.17)	8.8 (0.06)	14.9	-	7.2	7.8	-	N	N
014080	Toxofilin	5.6 (0.02)	8.6 (0.67)	8.3 (0.01)	6.6 (0.04)	8.8 (0.12)	12.9	-	9.8	4.5	-	Y	Y
030470	ROP46, putative	4.7 (0.22)	7.9 (0.18)	6.7 (0.01)	5.1 (0.20)	5.6 (0.03)	10.8	[2.9]	3.7	3.1	2.2	N	N
058370	ROP28	4.5 (0.06)	6.7 (0.36)	5.9 (0.00)	3.5 (0.08)	6.8 (0.22)	4.1	-	2.3	3.0	-	Y	N
108080	ROP5	4.7 (0.16)	8.5 (0.40)	7.8 (0.02)	6.7 (0.09)	5.5 (0.16)	20.0	-	9.7	2.7	6.5	Y	Y
065120	RON2L2 [Sporo-RON2]	3.7 (0.21)	6.2 (0.42)	5.5 (0.02)	3.4 (0.01)	3.7 (0.02)	3.6	-	2.1	2.3	2.1	Y	N
027810	ROP11	4.8 (0.14)	8.2 (0.57)	7.2 (0.04)	7.3 (0.01)	5.9 (0.08)	14.3	-	5.5	-	3.0	Y	Y
042110	ROP38 (ROP2L5)	4.7 (0.14)	6.0 (0.45)	6.5 (0.03)	6.0 (0.12)	5.1 (0.14)	2.1	-	3.2	-	2.6	N	N
015780	ROP2A (ROP2)	5.2 (0.19)	8.2 (0.58)	7.5 (0.12)	8.3 (0.02)	7.4 (0.17)	11.3	-	5.8	-	-	Y	Y
005250	ROP18	5.0 (0.39)	7.8 (0.52)	7.2 (0.09)	7.2 (0.10)	6.3 (0.15)	9.1	-	5.2	-	-	Y	Y
106060	RON8	5.6 (0.32)	7.7 (0.53)	7.0 (0.09)	7.2 (0.02)	6.5 (0.09)	5.4	-	3.0	-	-	Y	Y
115220	ROP14	4.4 (0.21)	6.5 (0.59)	6.2 (0.18)	6.4 (0.15)	6.3 (0.13)	3.5	-	2.8	-	-	Y	Y
109590	ROP1	4.5 (0.34)	6.7 (0.46)	6.2 (0.22)	7.4 (0.03)	5.5 (0.15)	3.9	-	2.6	-	-	Y	Y
058660	ROP6	4.8 (0.42)	7.0 (0.51)	6.3 (0.01)	6.9 (0.04)	6.1 (0.20)	4.7	-	2.5	-	-	Y	Y
011260	ROP26	4.8 (0.41)	6.2 (0.40)	5.9 (0.08)	7.0 (0.01)	7.5 (0.18)	2.3	-	-	-	[3.8]	N	Y
015770	ROP8	4.0 (0.04)	4.2 (0.67)	3.7 (0.04)	4.3 (0.12)	7.2 (0.12)	-	-	-	-	[7.8]	N	Y
003990	ROP12	4.5 (0.13)	6.4 (0.37)	5.4 (0.04)	6.5 (0.01)	6.1 (0.25)	3.2	-	-	[2.3]	-	Y	Y
058580	ROP17	4.9 (0.14)	7.0 (0.34)	6.4 (0.04)	7.5 (0.01)	6.7 (0.01)	4.3	-	2.7	[2.5]	-	Y	Y
053330	Rhoptry kinase family	3.9 (0.13)	2.8 (0.17)	2.9 (0.11)	5.8 (0.07)	8.3 (0.02)	[1.3]	-	[1.3]	[3.1]	[25.6]	N	Y
062050	ROP39	4.0 (0.17)	4.4 (0.22)	3.7 (0.03)	6.1 (0.03)	4.6 (0.20)	-	-	-	[3.2]	[1.3]	N	Y
042240	ROP19	4.0 (0.21)	5.2 (0.00)	4.6 (0.19)	7.2 (0.11)	5.0 (0.44)	-	-	-	[6.0]	-	N	N

Mean normalized, glog-transformed expression values and calculated fold-change in expression levels for each pair-wise comparison. The standard error of the mean (SEM) is given in parentheses.

1 TGME49_Gene identifier according to ToxoDB.org (v6.4).

2 Identity of protein assigned by ToxoDB.org (v6.4).

3 Oocysts sporulated for 0, 4 or 10 days.

4 *in vitro*-derived tachyzoites (2 dpi).

5 *in vivo*-derived bradyzoites (21 dpi).

6 Detection in day 10 (mature) oocyst proteome. Yes (Y) indicates that a minimum of 2 unique peptides mapping to given protein were identified by mass spectrometry.

7 Previous mass spectrometry evidence of expression in tachyzoites according to ToxoDB.org (v6.4).

*Fold change calculated from glog mean expression values back transformed to the original scale (see materials and methods) and shown only where values are significantly different, p<0.05.

**Rhoptry designation confirmed or putatively designated without confirmation of localization.

In tachyzoites, RON2 is known to directly interact with AMA1 during moving junction (MJ) formation and host cell invasion [54]–[56]. Both *AMA1* and *RON2* are expressed in d4 and d10 oocysts with transcript levels comparable to tachyzoites (Table 5 and Table S4) suggesting the usual tachyzoite-like MJ machinery operates in this stage. While the RON2 glog levels were relatively modest, in percentile terms they show expression ranging from 44^th^ percentile to 62^nd^ percentile in tachyzoites and d4 oocysts, respectively (as opposed to 9^th^ percentile for the d0 oocysts where their expression appears to be essentially off; Table S4). In addition, however, there was a specific up-regulation in d10 (and d4) oocysts of paralogous genes for both *RON2* (so-called “RON2-like2” or *RON2L2* in table 6 which we will hereafter refer to as “*SporoRON2*”) and *AMA1* (the “AMA1-paralogue” of Table 5 that we will hereafter refer to as “*SporoAMA1*”). Combined with the fact that the expression data for these two paralogues in both tachyzoites and bradyzoites was close to background levels (a glog expression value ≤3.7 for both genes in both asexual stages), these results strongly suggest that there exists a novel, sporozoite-specific alternative to the tachyzoite form of the MJ. Consistent with this, there was proteomic detection of both these novel paralogues in the d10 oocysts but no such evidence for expression in tachyzoites (Tables 5 and 6). A second *RON2*-paralogue, *RON2L1*, was detected in the oocyst, but not in the tachyzoite, proteome. Expression values for *RON2L1* were ∼5 across all oocyst, tachyzoite and bradyzoite samples, with no statistically significant difference in any comparison; therefore, it does not appear in Table 6. Of the other three components of the MJ, RON4, RON5 and RON8, only RON4 has a known paralogue, RON4L1. This gene's expression was significantly higher in bradyzoites than in d10 oocysts (∼2.7-fold), but did not statistically differ in other pair-wise comparisons. *RON4L1* expression was relatively low in day 10 oocysts (4.4), versus tachyzoites (5.3) and bradyzoites (6.2). In agreement with these transcript expression data, there is proteomic evidence of RON4L1 in tachyzoites, but not in oocysts. Hence, it would appear that the RON4L1 paralogue is not participating in the novel, sporozoite-specific MJ pairing with SporoRON2 and SporoAMA1 and that the rest of the MJ machinery (RON4/5/8) is also unvarying. Whether the SporoRON2/SporoAMA1 pair functions independently of the other RONs or in complex with them, like the normal RON2/AMA1 pair, is an important question for further investigation.

Dense granule proteins (GRAs) are secreted towards the end of parasite entry into the host cell and likely serve a role in maintenance of the parasite's intracellular niche within the parasitophorous vacuole, though their function is generally not well understood [60]–[62]. GRAs that have previously been identified in oocysts based on antibody detection include GRA1, GRA2, GRA4, GRA5, GRA6, GRA7 and GRA8 [21]. In this study, RNA levels for *GRA1–8* and *GRA14* increased in oocysts as they matured. Consistent with previous studies, *GRA3* and *NTPase* expression in d10 oocysts remained significantly lower than in both bradyzoites and tachyzoites [63]. *GRA8* expression was significantly higher in d10 oocysts compared to bradyzoites and did not differ significantly from tachyzoite levels. *GRA14* expression was significantly higher in d10 oocysts than in both tachyzoites and bradyzoites (Table 7). As before, the proteomic data strongly corroborated these results with all the above GRAs detected in tachyzoites and all but the three with glog expression values below 7 (GRA3, GRA9 and NTPaseI) detected in d10 oocysts.

**Table 7:** Functionally interesting genes of *Toxoplasma gondii* at different stages of oocyst maturation with a comparison to bradyzoite and tachyzoite expression levels - dense granules.

		Mean glog expression (SEM)					Fold-change * [fold-change lower]					Proteomic evidence	
Gene ID^1^	Product^2^	d0^3^	d4^3^	d10^3^	Tz^4^	Bz^5^	d4 vs. d0	d10 vs. d4	d10 vs. d0	d10 vs. Tz	d10 vs. Bz	Oo^6^	Tz^7^
039740	GRA14	5.2 (0.01)	9.3 (0.02)	8.9 (0.02)	7.6 (0.05)	6.8 (0.06)	33.6	[1.6]	21.7	3.4	6.7	Y	Y
075440	GRA6	4.8 (0.13)	8.1 (0.12)	8.3 (0.07)	7.8 (0.03)	7.1 (0.08)	12.2	-	15.3	1.7	3.0	Y	Y
054720	GRA8	4.8 (0.37)	8.6 (0.24)	8.9 (0.06)	8.3 (0.06)	5.7 (0.10)	20.1	-	27.2	-	15.9	Y	Y
003310	GRA7	4.9 (0.38)	9.5 (0.06)	9.6 (0.09)	8.7 (0.09)	8.2 (0.17)	45.9	-	53.4	-	4.0	Y	Y
110780	GRA4	4.8 (0.37)	6.8 (0.37)	7.1 (0.08)	7.0 (0.10)	5.9 (0.04)	3.9	-	5.1	-	2.8	Y	Y
070250	GRA1	5.3 (0.26)	10.0 (0.06)	9.7 (0.08)	9.1 (0.03)	9.3 (0.02)	61.2	-	46.9	-	-	Y	Y
086450	GRA5	5.5 (0.29)	9.6 (0.09)	9.5 (0.08)	9.1 (0.04)	9.3 (0.08)	35.6	-	32.9	-	-	Y	Y
027620	GRA2	4.8 (0.19)	8.3 (0.10)	8.3 (0.04)	9.2 (0.02)	8.8 (0.02)	14.6	-	15.5	[2.5]	-	Y	Y
027280	GRA3	5.0 (0.29)	5.2 (0.49)	6.7 (0.06)	8.3 (0.05)	8.1 (0.06)	-	2.9	3.2	[4.3]	[3.8]	N	Y
051540	GRA9	4.5 (0.23)	3.8 (0.03)	4.2 (0.06)	5.4 (0.03)	6.6 (0.05)	[1.3]	-	-	[1.7]	[4.3]	N	Y
077240	NTPaseI	4.0 (0.14)	4.0 (0.05)	4.2 (0.02)	7.6 (0.11)	4.7 (0.12)	-	-	-	[9.5]	-	N	Y

Mean normalized, glog-transformed expression values and calculated fold-change in expression levels for each pair-wise comparison. The standard error of the mean (SEM) is given in parentheses.

1 TGME49_Gene identifier according to ToxoDB.org (v6.4).

2 Identity of protein assigned by ToxoDB.org (v6.4).

3 Oocysts sporulated for 0, 4 or 10 days.

4 *in vitro*-derived tachyzoites (2 dpi).

5 *in vivo*-derived bradyzoites (21 dpi).

6 Detection in day 10 (mature) oocyst proteome. Yes (Y) indicates that a minimum of 2 unique peptides mapping to given protein were identified by mass spectrometry.

7 Previous mass spectrometry evidence of expression in tachyzoites according to ToxoDB.org (v6.4).

*Fold-change calculated from glog mean expression values back transformed to the original scale (see materials and methods) and shown only where values are significantly different, p<0.05.

### Antioxidant systems

*Toxoplasma* possesses a number of enzymes associated with detoxification of reactive oxygen species, such as superoxide dismutase (SOD), catalase, glutathione peroxidase, glutaredoxin, glutathione/thioredoxin peroxidase and peroxiredoxin [48]. Glutaredoxin and superoxide dismutase (*SOD3*) were included in the top 30 genes most abundantly expressed at the RNA level in d10 oocysts compared to tachyzoites and bradyzoites (Table 3); both have been shown previously to be present in sporulated oocysts [48] and both were detected in the d10 oocyst but not tachyzoite proteomes. In addition to the antioxidant enzymes, RNA corresponding to an oxidoreductase family protein, alanine dehydrogenase (TGME49_115260), was uniquely abundant in d10 oocysts compared to tachyzoites and bradyzoites with corresponding proteomic data to match (present in d10 oocysts but not tachyzoites; Table 3).

### Putative oocyst wall components

The wall compositions of two Apicomplexan genera, *Eimeria* and *Cryptosporidium*, have been partially characterized and serve as models for wall composition in *Toxoplasma*. In *Eimeria*, the oocyst wall is composed of proteins that are rich in tyrosine and undergo tyrosine-protein crosslinkages, providing structural robustness and resulting in the characteristic autofluorescence when exposed to UV light [64]. The genes for six tyrosine-rich proteins were markedly up regulated in the d10 oocysts compared to tachyzoites and bradyzoites where they appeared essentially off (with glog expression values generally below 5; Table 8). Interestingly, two of the tyrosine-rich proteins (TGME49_037080 and TGME49_087250) had peak expression levels in d0 oocysts as might be expected for proteins that are structural components of the oocyst wall. Expression of the remaining four tyrosine-rich genes peaked in d4 oocysts at the time the sporocyst walls are forming, suggesting that they may be being incorporated into the walls of the sporocysts, which are also autofluorescent and therefore might also contain dityrosine-protein crosslinkages as has been proposed for oocyst walls. All six of these tyrosine-rich proteins were detected in our proteomic analysis of oocysts. The two with the highest levels of expression observed in d0 oocysts were also enriched in the wall fractions, (TGME49_037080 and TGME49_087250), suggesting they are a part of the wall and not the sporocysts or sporozoites within. These tyrosine-rich proteins and their putative locations are described in more detail in the accompanying manuscript.

**Table 8:** Functionally interesting genes of *Toxoplasma gondii* oocysts at different stages of maturation with a comparison to bradyzoite and tachyzoite expression levels - other proteins of interest.

		Mean glog expression (SEM)					Fold-change * [fold-change lower]					Proteomic evidence	
Gene ID^1^	Product^2^	d0^3^	d4^3^	d10^3^	Tz^4^	Bz^5^	d4 vs. d0	d10 vs. d4	d10 vs. d0	d10 vs. Tz	d10 vs. Bz	Oo^6^	Tz^7^
**Tyrosine-rich proteins (% tyrosine)**													
037080	hypothetical protein (6.2%)	11.2 (0.52)	9.6 (0.35)	9.7 (0.05)	3.6 (0.22)	3.7 (0.03)	-	-	-	90.9	84.6	Y	N
120530	hypothetical protein (5.6%)	6.3 (0.13)	9.9 (0.15)	9.4 (0.07)	3.3 (0.20)	3.6 (0.20)	29.1	-	18.2	76.9	69.7	Y	N
081590	hypothetical protein (15.5%)	5.9 (0.01)	10.6 (0.07)	9.9 (0.08)	4.9 (0.04)	4.6 (0.28)	81.2	[2.15]	37.7	66.7	77.6	Y	N
087250	hypothetical protein (13.5%)	11.0 (0.44)	8.6 (0.73)	9.1 (0.02)	4.3 (0.01)	3.6 (0.09)	[10.6]	-	[6.3]	41.7	50.7	Y	N
119890	hypothetical protein (5.5%)	4.7 (0.29)	10.3 (0.04)	9.3 (0.04)	5.6 (0.13)	4.2 (0.04)	114.8	[2.78]	41.3	26.3	51.2	Y	N
116550	hypothetical protein (19.5%)	3.9 (0.12)	9.7 (0.04)	7.8 (0.13)	3.6 (0.13)	3.9 (0.14)	87.0	[6.77]	12.9	13.7	12.6	Y	N
**Putative oocyst wall proteins**													
004420	TgOWP1	5.1 (0.17)	7.4 (0.58)	7.4 (0.05)	4.3 (0.14)	5.0 (0.01)	5.5	-	5.6	8.2	56.0	N	N
009610	TgOWP2	10.7 (0.44)	8.4 (0.74)	8.8 (0.02)	3.3 (0.19)	3.9 (0.13)	[10.2]	-	[7.0]	38.5	34.0	Y	N
068310	TgOWP3	4.5 (0.02)	5.9 (0.47)	5.6 (0.00)	3.6 (0.08)	4.4 (0.15)	2.3	-	-	2.3	1.8	N	Y
022940	TgOWP4	4.4 (0.03)	6.7 (0.29)	5.9 (0.03)	4.8 (0.05)	4.5 (0.07)	4.3	[1.96]	2.20	1.9	2.1	N	N
048730	TgOWP5	5.4 (0.07)	7.6 (0.49)	7.5 (0.04)	3.9 (0.12)	4.6 (0.07)	6.2	-	5.44	10.0	7.8	N	N
010950	TgOWP7	3.7 (0.23)	5.8 (0.51)	5.6 (0.16)	3.5 (0.04)	3.7 (0.03)	2.5	-	2.35	2.5	2.3	N	N
**Late embryogenesis abundant domain-containing proteins (LEAs)**													
076850	LEA (TgERP)	4.9 (0.07)	10.4 (0.14)	9.4 (0.02)	3.5 (0.31)	4.4 (0.05)	110.7	-	44.7	71.4	55.4	Y	N
076880	LEA	4.3 (0.13)	10.2 (0.14)	9.3 (0.02)	3.5 (0.03)	3.8 (0.04)	123.7	[2.39]	51.7	66.7	60.0	Y	N
076860	LEA	4.1 (0.23)	9.3 (0.30)	8.0 (0.10)	3.3 (0.23)	3.8 (0.09)	52.2	[3.43]	15.2	18.9	16.4	Y	N
076870	LEA1 protein, putative	5.8 (0.46)	9.3 (0.06)	7.6 (0.02)	3.4 (0.09)	4.0 (0.24)	23.4	[5.47]	4.3	11.9	10.4	Y	N

Mean normalized, glog-transformed expression values and calculated fold-change in expression levels for each pair-wise comparison. The standard error of the mean (SEM) is given in parentheses.

1 TGME49_Gene identifier according to ToxoDB.org (v6.4).

2 Identity of protein assigned by ToxoDB.org (v6.4).

3 Oocysts sporulated for 0, 4 or 10 days.

4 *in vitro*-derived tachyzoites (2 dpi).

5 *in vivo*-derived bradyzoites (21 dpi).

6 Detection in day 10 (mature) oocyst proteome. Yes (Y) indicates that a minimum of 2 unique peptides mapping to given protein were identified by mass spectrometry.

7 Previous mass spectrometry evidence of expression in tachyzoites according to ToxoDB.org (v6.4).

*Fold change calculated from glog mean expression values back transformed to the original scale (see materials and methods) and shown only where values are significantly different, p<0.05.

Unlike *Eimeria* and *Toxoplasma*, *Cryptosporidium* oocyst walls do not autofluoresce and have a cysteine-rich wall that is thought to be strengthened by disulfide cross-linkages between the OWPs [65]. Six of the seven TgOWP homologues have corresponding probesets on the *T. gondii* array. In addition to the two discussed above (TgOWP2 and 5), all four of the remaining COWP homologues (TgOWP1, 3, 4 and 7) had significantly higher expression levels in d10 oocysts compared to both tachyzoites and bradyzoites (Table 8). Interestingly, however, only one of these proteins was detected in the proteomic analysis of d10 oocysts (TgOWP2) and a different one (TgOWP3) was detected in the tachyzoite proteome (Table 8), although this latter protein was found in only one of the five tachyzoite proteomic analyses and only two peptides were seen. TgOWP1, TgOWP2 and TgOWP3 have, however, all previously been detected using antibody reagents in *Toxoplasma* oocysts [45].

## Discussion

The results described here provide much information about the genes involved in oocyst development from the initial, relatively amorphous, immature form to the mature entity with its eight fully formed sporozoites. Several genes' transcripts were found to be most abundant in “d0” oocysts compared to later oocyst stages, tachyzoites and bradyzoites. These genes likely function in early oocyst development and initial sporozoite formation. Some, however, may represent genes that were involved in prior development within the feline enterocyte where the oocyst begins life; in these latter cases, the RNA detected may be residual. It is important to remember, in this context, that the sort of microarray analyses used here measure RNA abundance not transcription *per se* and so there can be a lag between detecting a given gene's transcripts and the actual time when that gene was being actively transcribed. Similarly, the “d0” sample was collected from feces on the first day that a substantial oocyst load was detected. These feces may have been shed up to 24 hours prior to the time when they were collected (the feces were collected at the same time each day). Hence, the very first few hours after initial shedding may not have been captured and some significant changes in the transcriptome may have occurred subsequent to the feces first emerging (e.g., due to changes in temperature and exposure to air).

Among the genes most abundantly expressed by d0 oocysts were two meiotic recombination genes encoding a DMC-like protein and a putative SP011. DMC and SP011 homologues are required for meiotic homologous recombination through chromosome alignment and double-strand breaks [66]. The precise sequencing of the steps in meiosis in *Toxoplasma* oocysts has not yet been explored but these data suggest the process is far from finished when oocysts are first shed. Transcripts for two scavenger receptor proteins (TgSR1 and SR2) and an LCCL-domain-containing protein (LCCL refers to ***L*** *imulus* clotting factor **C**, **C**och-5b2, **L**gl1 domain [67]) were uniquely abundant in d0 oocysts. In *Plasmodium berghei* the scavenger receptor protein PbSR is synthesized by macrogametes and is critical to sporogony; sporozoites fail to form in the oocysts of PbSR knockout parasites [68], [69]. Further, PbSR is a member of a family of LCCL proteins in *P. berghei* that appear to be structural paralogues involved in sporozoite development and infectivity. A putative major facilitator protein that has conserved regions with the Major Facilitator Superfamily (MFS), based on BLAST analysis of the predicted amino acid sequence, was up-regulated in d0 oocysts. The MFS comprises a diverse group of proteins, with at least 17 distinct families, generally involved in transport (uniport, symport or antiport) of small solutes in response to chemiosmotic gradients [70], [71]. A wide range of functions has been described for MFS proteins, including uptake of sugars [71] and efflux of drugs and metabolites [72]. Another gene with specifically elevated levels in the d0 oocyst is C-protein immunoglobulin A (IgA)-binding beta antigen. IgA-binding proteins have been described in pathogenic bacteria including group B *Streptococcus* where they are expressed as surface proteins that bind the Fc portion of human IgA and prevent their interactions with Fc receptors [73], thereby interfering with the effector function of host IgA [74]. IgA is critical to effective mucosal immune responses in the gut where it serves as a first line of defense against pathogens at mucosal surfaces [75]. It is possible that the C-protein IgA-binding beta antigen protein plays a role in mitigating host cell responses to the oocysts that are emerging from the feline enterocytes into the gut lumen for excretion into the environment. Alternatively, this protein's role might be in the gut of the intermediate host in which the sporozoites are initiating a new infection. Arguing against this latter notion is that this gene's expression declines later in oocyst development, when sporozoites are being actively generated.

Among the more interesting trends that emerged from our analyses of a time course of oocyst development is the remarkable predominance of ribosomal protein genes in the list of most up-regulated genes in d10 versus d4 oocysts (Fig. 2 and Table S3). While some ribosomal proteins are encoded by two or more paralogous genes, there was no trend for the genes observed as up-regulated to be paralogues of genes that were correspondingly down-regulated over the same time period. That is, there did not seem to be a d10 oocyst-specific set of ribosomal proteins that replaced a paralogous set expressed earlier. Instead it would seem that d10 oocysts are up-regulating ribosomal protein genes in general, perhaps to build a stock of ribosomes for the long period most oocysts must spend in the environment before being ingested. This store of ribosomes might enable an oocyst that suddenly finds itself in an intermediate host's intestine, after months or years of relative dormancy in the environment, to rapidly restart translation and development in order to launch a new infection.

The predominance of genes encoding secreted proteins among the set that is up-regulated in d4 and 10 oocysts, relative to d0, likely corresponds to the need for stocking the many secretory organelles that must be made, *de novo*, in the nascent sporozoites (rhoptries and micronemes, at least, are not recognizably present in the immature oocyst). The final, mature sporozoite must have a full complement of such proteins ready for the time when they encounter a new host and must invade. It is interesting that genes encoding several of the ROP proteins that are known to play a key role in co-opting host functions upon invasion by tachyzoites (ROP5, ROP16 and ROP18) are amply expressed in mature oocysts/sporozoites. This suggests that these protein kinases and pseudokinases may serve a similar function for the invading sporozoite as they do for the tachyzoites in which they were first discovered [76], [77]. A few *ROP* genes, e.g., *ROP42*, are substantially up-regulated in d10 oocysts relative to tachyzoites (although they are expressed in the latter at a low level). The proteins encoded by these genes may meet a special challenge for the invading sporozoite that tachyzoites do not face, perhaps related to invasion of gut epithelia which tachyzoites would not normally have an opportunity to invade. Interestingly, *ROP42* is also highly expressed at the RNA level in bradyzoites, consistent with a possible role for ROP42 in establishing an early infection in the gut since bradyzoites too must invade this tissue in order to start a new infection.

The function of the SRS family of surface proteins is largely unknown despite being one of if not the largest gene families in *Toxoplasma* (over 150 paralogous genes are present in the RH strain genome; [43]). The founding member of this family, SAG1, has been implicated in attachment of parasites to a host cell [78] and in somehow impacting the inflammatory response to the infection [79] but this does little to explain the pressures leading to the enormous gene expansion of this gene family. Genes for two SRS proteins were found to be strongly up-regulated in d10 oocysts relative to all other datasets in our analysis, the previously described “*SporoSAG*” and the gene encoding one of the original SRS proteins to be identified in tachyzoites and an important player in tachyzoite-mediated infection in mice, SAG3 [80]. Given their purported importance in the early stages of host cell invasion [81], it might have been expected that sporozoites and bradyzoites would share more similarity in their surface antigen repertoire (i.e., since they share a common gastrointestinal route of infection and therefore might encounter similar host cell receptors). It was surprising, therefore, that the opposite was seen: the oocysts more closely resembled tachyzoites than bradyzoites in their pattern of *SRS* gene expression. This added information about stage-specificity of their expression provides further clues to the still enigmatic function of SRS proteins.

One of the most striking findings in this work was the oocyst-specific expression of paralogues of AMA1 and RON2. AMA1 is expressed on the surface of tachyzoites where it binds to RON2 on the host cell surface; RON2 is injected into a host cell as one of the first steps in invasion and the parasite thereby provides its own receptor for attachment [54]–[56]. The fact that there are “Sporo” versions of these two key proteins suggests that they again meet a special need of sporozoites. Interestingly, neither protein has ever been detected within tachyzoites and the corresponding transcripts are likewise seemingly not expressed in tachyzoites and bradyzoites (there are no publicly available proteomic datasets for the latter stage). Whether this novel pair of proteins functions at the moving junction of sporozoites, or perhaps serves some new role, will be technically challenging to demonstrate as engineering parasites that express tagged versions of proteins and that can still go through the entire life cycle of *Toxoplasma* has never been reported. Unfortunately, an approach using polyclonal antibodies to these proteins is also likely to be problematic as these are likely to cross-react with their respective paralogues, which were also detected in the sporozoites. The final challenge will be catching a sporozoite mid-way during invasion, which is the only time that the moving junction exists; it is difficult to efficiently release sporozoites and follow their subsequent infection en masse. Such work will be important to attempt, however, as the original AMA1/RON2 pairing, at least, serves such a pivotal role in tachyzoites and this new pair seems likely to be serving an equally interesting and important role in sporozoites.

Once fully mature, the oocyst is generally considered to be inert and to exist in a dormant state in the environment until ingested by a susceptible intermediate host. Presumably it must therefore persist with its limited stores of energy such as the amylopectin granules that are present in the sporozoites [82]. Our data do not address what the transcriptome would look like in an oocyst that has persisted in the environment or under laboratory storage conditions for prolonged periods of time such as months or years. Such information will come from analysis of oocysts stored in conditions that mimic the natural environment (e.g., soil or water). Overall, the data presented here, however, present a detailed insight into the development of a previously mysterious stage in the *Toxoplasma* life cycle. They reveal a number of important differences from the asexual stages that are likely key to the unique role for this developmental form.

## Materials and Methods

### Ethics statement

All kitten and mouse experiments were conducted conforming to the guidelines of the Animal Welfare Act and the Health and Research Extension Act. Experimental protocols were approved by the UC Davis Institutional Animal Care and Use Committee, which is accredited by the Association for Assessment and Accreditation of Laboratory Animal Care International (IACUC #15619). Efforts were made to minimize the numbers of animals used to generate *Toxoplasma* organisms. The kittens used in the study remained healthy throughout. After two weeks of confirmed absence of shedding of *Toxoplasma* oocysts, the kittens were vaccinated and neutered, then adopted out to pre-screened and approved permanent homes.

### Toxoplasma gondii oocyst production

#### Mouse Infection

Initial mouse infections were done using *Toxoplasma gondii* oocysts produced in kittens in our laboratory using previously described methods [83]. The oocysts were from the M4 strain of *T. gondii,* isolated from an aborted sheep fetus and donated to our laboratory by the Moredun Research Institute of Scotland. Twenty 10-week old female Swiss Webster mice (Charles River Laboratories, Wilmington, MA) were inoculated subcutaneously (SQ) with 1,000 *Toxoplasma gondii* oocysts suspended in 200 µl phosphate buffered saline (PBS). One of the mice was given brain homogenate from a previously infected mouse, in addition to the oocysts SQ and a second mouse was given 1,000 oocysts *per os* (PO) by gastric gavage, in addition to the SQ oocyst inoculum. Mice were bled every two weeks, beginning 3 weeks after inoculation, to monitor *T. gondii* titers. At 6 weeks post-infection (wpi) 3 mice were euthanized for evaluation of bradyzoite brain cyst formation by histology. Eight weeks after infection 10 mice were sacrificed and their brains collected. Half of each brain was fed to the kittens and the other half was submitted for histology for verification of status and rate of infection.

#### Kitten Infection

To produce oocysts for these experiments two 12-week-old specific-pathogen-free kittens (Nutrition and Pet Care Center, Department of Molecular Biosciences, University of California, Davis) were infected by feeding a total of 2.5 mouse brains each (half of 5 brains each, as above). An indirect fluorescent-antibody test (IFAT) was performed to ensure that they were seronegative for *Toxoplasma gondii* antibodies prior to infection.

#### Oocyst Harvest from Kitten Feces

Feces were collected from kittens daily and examined by zinc sulfate double centrifugation to detect shedding of oocysts as well as monitor for co-infection with other parasites. Kittens were shedding *Cryptosporidium* oocysts prior to infection with *T. gondii*. However, *Cryptosporidium* oocysts were not detected after shedding of *T. gondii* oocysts commenced. No other protozoal organisms were observed. Once *T. gondii* oocysts were detected in feces, all procedures were conducted in a biohazard hood and unsporulated oocysts were harvested from feces using sodium chloride (specific gravity 1.20) to concentrate the oocysts by flotation. Following the final wash step, the resultant oocyst pellet was resuspended in approximately 12 ml of 2% sulfuric acid and transferred to a T75 tissue culture flask for sporulation. Oocysts were incubated and aerated by gentle rocking for a defined period (4 days or 10 days) at room temperature (∼22°C) to allow for sporulation. Day 0 oocysts (0% sporulated) were collected prior to aeration in sulfuric acid and directly purified.

#### Oocyst Purification

Prior to purification, oocysts were washed three times in PBS to remove sulfuric acid and restore neutral pH. Gradient separation was performed with CsCl in Tris-EDTA buffer (TE buffer), layering CsCl at specific gravities of 1.15, 1.10, 1.05 and oocysts in TE buffer as top layer, as previously described [84]. The specific gravity of *T. gondii* oocysts is between 1.05–1.10. Gradient preparations were performed in 50 ml polypropylene tubes. Samples were centrifuged at 16,000× g for 60 min. Oocysts were harvested at the 1.05/1.10 interface. The harvested layers were washed twice with PBS, using spins at 2500× g for 15 min each. The final pellet was resuspended in PBS.

### RNA from oocysts, tachyzoites and bradyzoites

#### Oocyst preparation for RNA extraction

5×10^6^ oocysts were harvested at each duplicate time point, suspended in 125 µL PBS and stored at −80°C until extracted. Upon extraction, frozen oocysts were re-suspended in 1 ml TRIzol (Invitrogen). After loading 1 ml TRIzol into the chamber of a pre-cooled (−80°C) French Pressure Cell (Thermo Electron Aminco French Pressure Cell, Model FA-003), the 1 ml oocyst suspension was added to the pressure cell chamber. The resulting 2 ml volume was pressed at 20,000 p.s.i. and a roughly 1.5 ml fraction was collected. Note: It was determined that approximately 0.5 ml was lost in the pressure cell, therefore 1 ml TRIzol was preloaded to occupy the dead space volume that would not be recovered. The pressure cell was sterilized between each sample by autoclaving then washing with Milli-Q water.

#### Tachyzoite preparation for RNA extraction

Tachyzoites of the same M4 isolate were grown in confluent monolayers of primary human foreskin fibroblasts (HFF) in DMEM (Invitrogen, Carlsbad, CA) with 10% fetal calf serum (FCS; Hyclone, Logan, UT), 2 mM glutamine, 100 U/ml and 100 ug/ml streptomycin at 37°C with 5% CO2. *In vitro* 2 dpi tachyzoite samples were collected from separately infected cultures of HFFs (replicate flasks were infected in parallel with an MOI of 3). Samples were harvested separately and processed independently for all steps. To isolate the parasites, HFFs were lysed by passage through a 27-gauge needle at least 10 times. Whole cells were pelleted by brief centrifugation (∼3 minutes) in a Sorvall RT7 plus tabletop centrifuge at 700 rpm (102× g). The parasites were collected by centrifugation of the supernatant at 1500 rpm (470× g) for 10 minutes. Parasites were then brought up in 1 ml TRIzol reagent (Invitrogen) and frozen at −80°C.

#### Bradyzoite cyst preparation for RNA extraction

Bradyzoite cysts were produced and isolated as previously described [83]. For biologic duplicates, two separate groups of four 8-week-old Swiss Webster mice were infected with 1,000 oocysts PO. One mouse in the first group was infected with 1,000 oocysts SQ. To minimize morbidity and prevent death in infected mice, all infected mice were treated with sulfadiazene (0.4 µg/ml in drinking water) for 10 days, beginning 10 days post-inoculation. Three weeks post-inoculation mice were sacrificed and brains were harvested.

The methods used to isolate bradyzoite cysts from mouse brains were modified from a previously described protocol [85]. Each brain was passed through a 100 µm cell strainer into a 50 ml conical tube using the plunger of a 6 ml syringe to press the tissue through the strainer and washing with PBS to a total volume of 4 ml. The brain suspension was then syringe-passed through a 16 gauge blunt needle 10 times followed by a 22 gauge blunt needle 10 times. The brain suspension was brought up to a total volume of 10 ml with PBS. A density gradient was prepared for each sample by carefully layering (from bottom to top) 9 ml 90% Percoll, followed 9 ml 30% Percoll, then 10 ml brain suspension in a 50 ml conical tube. Percoll dilutions were made using 1× PBS. Each gradient was centrifuged at 1,200× g for 15 minutes at 4°C. Cysts were harvested from the 30% and 30%/90% interface. Cyst suspensions were washed with 45 ml PBS and centrifuging at 1,500× g for 15 min at 4°C. The supernatant was removed to about 5 ml and the pellets were combined into one 50 ml tube. A second wash in PBS was performed by bringing the combined suspension up to 45 ml with PBS and centrifuging at 2,500× g for 15 minutes at 4°C. The supernatant was removed and the remaining pellet was transferred to a 1.5 ml microcentrifuge tube and brought up to 1 ml with PBS. A 10 µl aliquot was removed for cyst enumeration. The suspension was then centrifuged at 13,200 rpm for 8 minutes and the supernatant was removed. Cysts were counted by removing 10 µl of final cyst suspension to a glass slide with a coverslip. The entire area under the converslip was counted and the total estimated cyst number calculated. The final cyst pellet was resuspended in 1 ml TRIzol and stored at −80°C until RNA was extracted.

#### RNA Extraction and Microarray

RNA extraction methods were adapted from Invitrogen TRIzol instructions for RNA isolation, with a few modifications. Frozen samples were thawed in a 37°C water bath and then allowed to equilibrate to room temperature. 0.2 ml chloroform was added to TRIzol suspensions. Tubes were mixed by hand for 15 seconds then incubated for 3 minutes at room temperature. Tubes were then spun at 12,000 rpm for 20 minutes at 4°C. The aqueous phase containing the RNA was transferred into a fresh tube (∼550 µl). 0.5 ml isopropyl alcohol was added. Tubes were mixed by hand and incubated at room temperature for 10 minutes. Tubes were then spun at 13,000 rpm for 20 minutes at 4°C. To wash the RNA, supernatant was removed and 1 ml 75% ethanol was added to pellet. Tubes were inverted to mix by hand. Tubes were spun at 13,000 rpm for 20 minutes at 4°C. Supernatant was removed and the RNA pellet was air-dried in open tube for approximately 10 minutes. RNA was re-dissolved in 12 µl RNase-free water.

RNA preparation for microarray was conducted using the protocol and reagents provided in the Invitrogen Gene Chip 3′IVT Express Kit. 250 ng of total RNA was used as the starting material. Instructions for 169/400/HT format were followed for fragmentation and labeling of aRNA.

Samples were hybridized to the *Toxoplasma gondii* Affymetrix Array (Tgondiia520372) by the Stanford Protein and Nucleic Acid Facility. The following equipment was used to scan the arrays and generate. cel files: Affymetrix GeneChip Hybridization Oven 640, Affymetrix GeneChip Fluidics Station 450, Affymetrix GeneChip Scanner 3000 7G and Affymetrix Genechip Command Console (AGCC). AGCC was used to generate and normalize gene expression values.

### Microarray Analysis

#### Preprocessing

Data were converted from. cel files and averaged across probes within each probeset using the Bioconductor package affy (version 1.22.1, [86]) within the statistical software system R (version 2.10.1, [87]), and transformed via a generalized logarithm transformation [34], [88] using Bioconductor package LMGene (version 2.4.0, [89]).

#### Statistical Analysis

A one-way analysis of variance (ANOVA) model was fitted to the data one probeset at a time. The ANOVA model included a single factor for time/lifestage, with the following levels: Day 0 oocysts, Day 4 oocysts, Day 10 oocysts, tachyzoites, and bradyzoites. For probes for which the global F test of a time/lifestage effect was significant at the 5% level, indicating significant differences between at least two levels of the factor, Tukey HSD post-hoc tests were conducted to test for significant differences among the comparisons of interest (day 4 oocysts vs. day 0 oocysts, day 10 oocysts vs. day 4 oocysts, day 10 vs. day 0 oocysts, day 10 oocysts vs. tachyzoites, and day 10 oocysts vs. bradyzoites). For each of the above comparisons of interest, the statistical analysis produced a list of probesets/*Toxoplasma* gene IDs for which the expression levels differed significantly (Tukey HSD p<0.05) between the times/lifestages being compared, (due to the presence of controls, etc., not every probeset had a corresponding gene ID). Fold changes were calculated as follows: On the scale of the transformed data, the mean expression was calculated for each stage/sample type of interest (e.g. d0 oocyst). The mean for each was then transformed back to the original scale of the data by inverting the glog transformation, and the fold change was calculated as the ratio of back-transformed means.

#### Data Deposition

All data is MIAME compliant and the raw data have been deposited on the Gene Expression Omnibus (GEO) site (www.ncbi.nlm.nih.gov/geo/), GEO accession number: GSE32427. The complete searchable dataset is also available at ToxoDB.org.

## Supporting Information

Table S1 **Percentiles for glog expression values across dataset.**
(DOCX)

Table S2 **All probesets for which a significant difference (p<0.05) was identified in any of the following pair-wise comparisons: oocyst time points - d4 vs. d0, d10 vs. d0, d10 vs. d4; and between oocysts and asexual lifestages - d10 vs. tachyzoite (2 dpi), d10 vs. bradyzoite (21 dpi).**
(XLSX)

Table S3 **Lists of the top 30 significant genes (p<0.05) in each of the oocyst time point comparisons, listed in order of genes with the largest fold-change in mean expression level in each direction, up or down.**
(XLSX)

Table S4 **Summary of RON2/AMA1 and SporoRON2/SporoAMA1 expression across all samples.**
(DOCX)
