# Supplementary material for: Transcriptomic Analysis of Toxoplasma Development Reveals Many Novel Functions and Structures Specific to Sporozoites and Oocysts
Source: PLoS One. 2012 Feb 13;7(2):e29998. doi: 10.1371/journal.pone.0029998 (PMC3278417; doi:10.1371/journal.pone.0029998)
Supplement: Table S1 — Percentiles for glog expression values across dataset. (DOCX) [file pone.0029998.s001.docx]

| **Percentile** | **glog^1^** |
| --- | --- |
| 10th | 3.3 |
| 20th | 3.5 |
| 25th | 3.6 |
| 30th | 3.7 |
| 40th | 3.9 |
| 50th | 4.1 |
| 60th | 4.3 |
| 70th | 4.6 |
| 75th | 4.8 |
| 80th | 5.1 |
| 90th | 5.9 |
| 95th | 6.8 |
| 99th | 8.7 |
| 99.99th | 11.4 |

**Supplemental Table 1**. Percentiles for glog expression values across dataset

Percentiles for corresponding glog values calculated from probesets (controls excluded) across all sample groups.

For example, a glog value of 4.8 (75^th^ percentile) is higher than 75% of all other glog values in dataset.

^1^ Mean normalized, glog-transformed expression value
