# Supplementary material for: Transcriptomic Analysis of Toxoplasma Development Reveals Many Novel Functions and Structures Specific to Sporozoites and Oocysts
Source: PLoS One. 2012 Feb 13;7(2):e29998. doi: 10.1371/journal.pone.0029998 (PMC3278417; doi:10.1371/journal.pone.0029998)
Supplement: Table S4 — Summary of RON2/AMA1 and SporoRON2/SporoAMA1 expression across all samples. (DOCX) [file pone.0029998.s004.docx]

**Supplemental Table 4.** Summary of RON2/AMA1 and SporoRON2/SporoAMA1 expression across samples

|  | **RON2** | | **AMA1** | | **SporoRON2** | | **SporoAMA1** | |
| --- | --- | --- | --- | --- | --- | --- | --- | --- |
|  | **glog^1^** | **Percentile^2^** | **glog^1^** | **Percentile^2^** | **glog^1^** | **Percentile^2^** | **glog^1^** | **Percentile^2^** |
| **d0^3^** | 3.3 | 9 | 4.8 | 73 | 3.7 | 28 | 3.8 | 34 |
| **d4^3^** | 4.4 | 62 | 7.7 | 97 | 6.2 | 92 | 6.1 | 91 |
| **d10^3^** | 4.0 | 44 | 7.3 | 96 | 5.5 | 85 | 5.1 | 79 |
| **Tz^4^** | 4.2 | 54 | 7.1 | 96 | 3.4 | 12 | 3.6 | 22 |
| **Bz^5^** | 3.8 | 34 | 6.1 | 91 | 3.7 | 28 | 3.6 | 22 |

^1^ Mean normalized, glog-transformed expression value

^2^ Percentile of expression for corresponding glog value

^3^ Oocysts sporulated for 0, 4 or 10 days

^4^ *in vitro*-derived tachyzoites (2 dpi)

^5^ *in vivo*-derived bradyzoites (21 dpi)
